# Some comments on Bitcoin market (in)efficiency

**DOI:** 10.1371/journal.pone.0219243

**Published:** 2019-07-08

**Authors:** V. Dimitrova, M. Fernández-Martínez, M. A. Sánchez-Granero, J. E. Trinidad Segovia

**Affiliations:** 1 Department of Economics and Business, Universidad de Almería, Almería, Spain; 2 University Centre of Defence at the Spanish Air Force Academy, MDE-UPCT, Santiago de la Ribera, Región de Murcia, Spain; 3 Department of Mathematics, Universidad de Almería, Almería, Spain; Universidad Veracruzana, MEXICO

## Abstract

In this paper, we explore the (in)efficiency of the continuum Bitcoin-USD market in the period ranging from mid 2010 to early 2019. To deal with, we dynamically analyse the evolution of the self-similarity exponent of Bitcoin-USD daily returns via accurate FD4 approach by a 512 day sliding window with overlapping data. Further, we define the *memory* indicator by the difference between the self-similarity exponent of Bitcoin-USD series and the self-similarity index of its shuffled series. We also carry out additional analyses via FD4 approach by sliding windows of sizes equal to 64, 128, 256, and 1024 days, and also via FD algorithm for values of *q* equal to 1 and 2 (and sliding windows equal to 512 days). Moreover, we explored the evolution of the self-similarity exponent of actual S&P500 series via FD4 algorithm by sliding windows of sizes equal to 256 and 512 days. In all the cases, the obtained results were found to be similar to our first analysis. We conclude that the self-similarity exponent of the BTC-USD (resp., S&P500) series stands above 0.5. However, this is not due to the presence of significant memory in the series but to its underlying distribution. In fact, it holds that the self-similarity exponent of BTC-USD (resp., S&P500) series is similar or lower than the self-similarity index of a random series with the same distribution. As such, several periods with significant antipersistent memory in BTC-USD (resp., S&P500) series are distinguished.

## Introduction

The theoretical development of Efficient Market Hypothesis (EMH, hereafter) are attributed to both Cootner [1] and Samuelson [2]. One of the pioneers in the analysis of market efficiency was Fama [3], who considered that a market is efficient provided that all the information available is fully reflected in the market and can be used for all the agents. The market efficiency was classified into three states, namely, weak efficiency, when market prices do reflect all the information contained in the past series of prices, semi-strong efficiency, if prices reflect all the public information, and strong efficiency, as well. In this way, that last one becomes the most restrictive scenario since it considers that prices reflect all the public and private informations.

From a statistical viewpoint, an efficient market follows a random walk, first introduced in finance by the mathematician Bachelier to study the behavior of the French Bond prices [4]. As such, market prices would be described by independent and identically distributed random variables. The presence of long memory in series of prices or asset returns cannot be accepted in that scenario since it would allow a riskless profitable trading strategy.

Since its foundation, EMH has been questioned for being too restrictive and generic. In this way, several alternatives have been posed. The Mandelbrot’s proposal [5], where stock prices are fitted by a fractional Brownian motion, represents one of the first critics to EMH. Such a model assumes that prices exhibit long memory, which is not possible under EMH. In a recent contibution, Ponta and Carbone [6] remark the importance of market heterogeneity as a weak point of EMH, which is based on a homogeneous random process. They propose a cumulative Market Heterogeneity Index based on previous works (c.f. [7–9]), is related to the concept of entropy, and provides a better evaluation of portfolio composition in comparison with traditional Sharpe Ratio. This new approach takes into account the existence of clusters in financial data. Since Philippatos and Wilson [10] introduced the concept of entropy in finance, many other researchers have enriched the market finance theory by different entropy concepts to measure risk and describing distributions (see Zhou et al. [11] for a detailed review).

To explore the presence of long memory in stock prices or asset returns have become a discussing topic for market efficiency analysis. It is worth mentioning that a wide amount of papers do throw some empirical evidence of long memory (c.f., e.g., [12–19]), whereas others do not [20, 21]).

However, a few questions should be still addressed regarding the use of the self-similarity exponent to analyze EMH. For instance, several authors have recently suggested a relationship between the degree of development of a market and its level of efficiency (c.f., e.g., [22–25]). They assume that it may be quantified throughout its self-similarity exponent. As such, they analyze scaling behavior patterns to quantify their level of development.

As a consequence, they highlight that mature markets usually display short memory or no memory at all, whereas emerging markets still exhibit long memory properties. By the other hand, it is also frequent to extract conclusions of a simple analysis of the self- similarity index without addressing some relevant questions, such us, the underlying distribution of data (e.g. [26]) or the accuracy of different algorithms used to obtain *H* value (e.g. [27]). According with this, we consider an interesting issue the analysis of the Hurst exponent of the cryptocurrencies with the objetive of characterizing the degree of development of this new market. Along this paper, we shall be focused on the study of self-similarity patterns in the continuum Bitcoin-USD (BTC-USD, hereafter) evolution through time.

The present article is organized as follows. Firstly, Section 1 contains a detailed literature review of the main issues that have been addressed so far regarding the Bitcoin maket behavior. In Section 2, we provide the basics on FD4 algorithm applied along this paper for self-similarity exponent calculation purposes. In Section 3, we present the results we obtained concerning the (in)efficiency of BTC-USD evolution through time, and finally, Section 4 highlights our main conclusions.

## 1 Cryptocurrencies: A literature review

Bitcoin was introduced on October 31st, 2008, through a paper, released to a few cryptography enthusiasts [28]. That email, signed by the pseudonym of Satoshi Nakamoto, explained that it was a cryptocurrency allowing exchange of value tokens between two parts without divulging any transaction details. Since then, Bitcoin has emerged as the most popular and demanded cryptocurrency.

Actually, Bitcoin is the cryptocurrency with the highest market capitalization among more than 1658 digital currencies existing at present, reaching 237.62 billion USD in December 2017. Since its appearance, its market capitalization has increased from approximately 0.04 billion USD in the first quarter of 2012 to 117.56 billion USD in the first quarter of 2018, according to [29].

The lack of control from governments and central authorities, the poor and inefficient regulation, and the youth of the cryptocurrencies have made their markets quite speculative and volatile. Despite this, the growing market of these new successful financial instruments and their innovative features attract more and more big and small investors, speculators, policymakers, and academic researchers from around the world.

The market of cryptocurrencies is still tiny compared to traditional financial markets. Therefore, there are only few relevant researches in the literature. It is worth pointing out that EMH has been analysed in cryptocurrency markets since 2015. Most of the researchers have been focused on weak efficiency throughout different approaches. Next, we shall comment the methodologies as well as the results contributed in some papers already appeared in the literature.

In [30], Bartos concentrated efforts to throw some evidence of efficiency in the Bitcoin market and explored the behavior of its price evolution by carrying out an empirical analysis. The results contributed therein suggest that such a cryptocurrency follows EMH and immediately reacts to publicly announced information. It was found that events affect prices of cryptocurrencies. More specifically, Bitcoin’s price stands higher (resp., lower) during days of positive (resp., negative) events than during days without remarkable events. Also, it was concluded that both demand and supply factors have a crucial impact on the price of that cryptocurrency.

Subsequently, Urquhart [31] investigated the efficiency of the Bitcoin market from August 1st, 2010 to July 31st, 2016, using five different tests on Bitcoin returns, namely, Ljung-Box, runs, Bartel’s, variance ratio, wild-bootstrapped AVR, and BDS. That research threw some empirical evidence against EMH regarding the Bitcoin evolution through time. In fact, it was concluded that the inefficiency of Bitcoin market is quite strong. However, it has to be mentioned that the whole time period was splitted into two subperiods, from August 1st, 2013 to July 31st, 2016, with the Bitcoin market being efficient only in the second one. As such, it was stated that it becomes more efficient with time.

After a while, Nadarajah and Chu [32], following up Urquhart [31], reexamined the data using eight different tests by adding odd integer power transformations of Bitcoin’s daily returns. The approaches they applied were Ljung-Box test (for no autocorrelation), runs and Bartel’s tests (for independence), wildbootstrapped automatic variance ratio and spectral shape tests (for the random walk hypothesis), BDS test (to guarantee that the returns are independently and identically distributed), robustified portmanteau test (for no serial correlation), and the generalized spectral test (for the martingale difference hypothesis). According to the results provided, there was no evidence against the null hypothesis, except the tests for independence. As such, the authors concluded that the Bitcoin market behaves efficiently.

Later, Kurihara and Fukushima [33] carried out an empirical analysis of Bitcoin’s market by checking its efficiency and looking for possible anomalies in its weekly prices. As a result, they stated that Bitcoin market is not efficient though will behave more efficiently over time. In this way, they suggested that it will be random in the future.

On the other hand, Bariviera, Basgall, Hasperué, and Naiouf [34] compared the Bitcoin’s dynamics to standard currencies’ and were focused on the analysis of returns at different time scales. They tested the presence of long memory in Bitcoin returns from 2011 to 2017 by using the Hurst exponent via the Detrended Fluctuation Analysis (DFA) over a sliding window to measure long range dependence. They also carried out a multi-scale analysis leading to similar results from the viewpoint of the Hurst exponents. As such, they detected that Hurst exponents changed significantly during the first years of existence of Bitcoin, tending to stabilize in recent times. More specifically, they stated that the Bitcoin series had a persistent behavior (a self-similarity exponent greater than 0.5) until 2014, whereas after that year, the Hurst exponent tended to move around 0.5. Accordingly, the Bitcoin market behaves more efficiently since 2014 with its behavior across different time scales (5 − 12 h) being essentially similar in terms of long memory.

Shortly afterward, Bariviera [35] reexamined the fluctuations of Bitcoin prices. On this occasion, he studied the time varying behavior of long memory for Bitcoin volatility and returns since 2011 to 2017. With this aim, they were applied both the R/S analysis (to detect long memory) and the DFA (to discriminate more precisely variations in informational efficiency across time) for Hurst exponent calculation purposes. Following the results, it was stated that the daily returns exhibit persistent behavior from 2011 until 2014, whereas the market became more informational efficient since 2014. However, the price volatility exhibits long memory along the whole time period.

Alvarez-Ramirez, Rodriguez, and Ibarra-Valdez [36], on their part, studied the presence of long-range correlations and informational efficiency of Bitcoin market for the period ranging from June 30th, 2013 to June 3rd, 2017, via DFA approach over sliding windows to estimate long-range correlations for Bitcoin price returns. The results obtained therein were similar to Bariviera’s: they revealed that the Bitcoin market exhibits periods of efficiency, alternating with periods where the price dynamics are driven by anti-persistence.

Tiwari, Jana, Das, and Roubaud [37] also tested the informational efficiency of Bitcoin. To that end, they used a battery of computationally efficient long-range dependence estimators for a period spanning from July 18th, 2010 to June 16th, 2017. The conclusions of their study indicated that the market is informational efficient as consistent to recent findings of Urquhart [31], Nadarajah and Chu [32], and Bariviera [35]. The authors emphasized that Bitcoin market is efficient with some exception to the period of April-August, 2013 and August-November, 2016.

Recently, Juang, Nie, and Ruan [38] investigated the time-varying long-term memory in the Bitcoin market through a rolling window approach and employing a new Efficiency Index [39], using daily datasets for the period from 2010 to 2017. They concluded that the generalized Hurst exponents in the Bitcoin market are above 0.5. From their point of view, long-term memory exists in Bitcoin market. They also observed a high degree of inefficiency in such a market and stated that it does not become more efficient over time.

At the beginning of 2018, Demir, Gozgor, Lau, and Vigne [40] published a paper which aimed to analyze the prediction power of economic policy uncertainty (EPU) index on the daily Bitcoin returns for the period from July 18th, 2010 to November 15th, 2017, via the Bayesian Graphical Structural Vector Autoregressive model, the ordinary Least Squares, and the Quantile-on-Quantile Regression estimations. The authors deduced that Bitcoin returns are negatively associated with changes in the EPU, but they also pointed out that the effect is positive and significant at lower and higher quantiles of Bitcoin returns and EPU.

Extending the Bitcoin market investigation, Brauneis and Mestel [41] linked efficiency to measures of liquidity. After performing some tests for normality (Jarque-Bera and Kolmogorov-Smirnov), the Engle’s ARCH test, the five tests applied by Urquhart (Ljung and Box, runs, variance ratio (VR), Kim’s wild bootstrapped VR, Bartel’s, and Brock et al. non-parametric BDS), the Hurst exponent, and non-parametric test for marketing efficiency on 73 cryptocurrencies in the period from August 31st, 2015 to November 30th, 2017, they concluded that cryptocurrencies become less inefficient as liquidity increases.

Furthermore, Gaporale, Gil-Alana, and Plastun [42] used R/S analysis and fractional integration long-memory techniques to examine the degree of persistence of Bitcoin, Litecoin, Ripple, and Dash cryptocurrencies over the sample period 2013 − 2017. In their opinion, there is evidence of market inefficiency since these markets exhibit persistence. In particular, they insist that there are positive correlations between their past and future values which change over time. From their point of view, the cryptocurrency market is still inefficient, but it is becoming less so, especially, in the case of Litecoin market, where the Hurst exponent dropped considerably over time.

In addition, Cheap, Mishra, and Zhang [43] proposed a new mechanism to understand dynamic interdependence of Bitcoin prices in a cross-market context. They modeled cross-market Bitcoin prices as long-memory processes and studied dynamic interdependence in a fractionally cointegrated VAR framework. As a result, long-memory was found in both, the individual markets and the system of markets depicting non-homogeneous informational inefficiency.

Kristoufek [44] recently published a paper on the study of efficiency of Bitcoin market with respect to both USD and Chinese yuan currencies, and their evolution over time. He used the Efficiency Index [23], for testing them for different types of (in)efficiency measures. As regards the USD market, he commented that there are only two longer periods of time when the market can be considered as efficient—from the middle of 2011 to the middle of 2012, and between March and November of 2014. Aside from them, there is no efficiency in the Bitcoin market. It is worth mentioning that the results of the analyses carried out regarding the CNY market are not so strong since the examination period misses some very important bubble-like dynamics before 2014. Therefore, he insists there is strong evidence that both Bitcoin markets remain mostly inefficient between 2010 and 2017, except several periods directly connected with cooling down after the bubble-like price surges.

Khuntia and Pattanayak [45] evaluated the adaptive market hypothesis (AMH) as well as the evolving return predictability in Bitcoin market, using two robust methods in a rolling-window framework to capture time-varying linear and nonlinear dependence in Bitcoin returns. The conclusions of their study are that efficiency of Bitcoin market evolves with time and the evidence of its dynamics adheres to the AMH. According to the authors, some crucial events coincide with episodes of (in)efficiency, so creation of events and behavioral bias may change its efficiency.

Moreover, Vidal-Tomás and Ibañez [46] evaluated the hypothesis of semi-strong efficiency of Bitcoin in both Bitstamp and Mt.Gox markets by applying the methodology of the event study. They wanted to check how digital currencies respond to monetary policy and Bitcoin events. The authors observed that Bitcoin has become more efficient over time in relation to its own events, but at the same time, they concluded that the cryptocurrency is not affected by monetary policy news, highlighting the absence of any kind of control on Bitcoin.

Nevertheless, the dynamic behavior of the cryptocurrencies has not been practically explored and the studies that have been carried out on these issues are still scarce. This is one of the reasons for the choice of analysis of the memory in the Bitcoin market as a subject of our enquiry. We consider important to identify the relationship existing between the Bitcoin market and the standard financial markets. As such, to dynamically test for long memory patterns through time in BTC-USD return series, we shall apply the so-called FD4 approach to accurately calculate the self-similarity exponent of (financial) time series.

## 2 FD4 approach

In this section, we shall provide the basics on the so-called FD4 algorithm, a procedure first introduced in [47, Sections 3 and 4] to accurately calculate the self-similarity exponent.

Let *q* > 0, *m_q_*(*X*) = E[|*X*|*^q^*] (provided that it exists), assume that **X** is a random process with stationary increments, and there exists *H* > 0 such that the next power law stands:

$$\begin{array}{c} M\left(T,\omega \right)∼T^{H}\cdot M\left(1,\omega \right), \end{array}$$ (1)

where M(t,T,ω) denotes the cumulative range of period T, i.e.,

$$\begin{array}{c} M\left(t,T,\omega \right)=\underset{t\leq s\leq t+T}{\sup}\left\{X\left(s,\omega \right)-X\left(t,\omega \right)\right\}-\underset{t\leq s\leq t+T}{\inf}\left\{X\left(s,\omega \right)-X\left(t,\omega \right)\right\} \end{array}$$

with M(T,ω)=M(0,T,ω). It is worth mentioning that Eq (1) is satisfied by any random function with self-affine increments of parameter *H*. A sufficient condition leading to this kind of random processes stands for self-similar processes with stationary increments (c.f. [48, Lemma 3.4]). The reciprocal is also true for random processes with self-affine increments.

Let Eq (1) be raised to the *q*−power and discretize the period T by $T_{n}=2^{-n}$. Thus,

$$\begin{array}{c} M{\left(T_{n},\omega \right)}^{q}∼T_{n}^{qH}\cdot M{\left(1,\omega \right)}^{q}. \end{array}$$ (2)

If we denote $X_{n}=M(T_{n},\omega )=M(2^{-n},\omega )$, then Eq (2) can be rewritten in the following terms:

$$\begin{array}{c} X_{n}^{q}∼T_{n}^{qH}\cdot X_{0}^{q} \end{array}$$

for all *q* > 0 and n∈ℕ. As such, the *q*−powers of cumulative ranges from consecutive periods of **X** are related throughout the following expression:

$$\begin{array}{c} X_{n}^{q}∼2^{qH}\cdot X_{n+1}^{q}. \end{array}$$ (3)

Hence, if the means of the random variables involved in Eq (3) exist, then it holds that $\text{E}[X_{n}^{q}]=2^{qH}\cdot \text{E}[X_{n+1}^{q}]$, or equivalently,

$$\begin{array}{c} m_{q}\left(X_{n}\right)=2^{qH}\cdot m_{q}\left(X_{n+1}\right), \end{array}$$ (4)

which throws a strong relationship between consecutive moments of order *q* for each n∈ℕ. Another option is to rewrite Eq (4) as follows:

$$\begin{array}{c} m_{q}\left(X_{n}\right)=\frac{1}{2^{nqH}}\cdot m_{q}\left(X_{0}\right). \end{array}$$ (5)

Let us apply 2−base logarithms to both sides of Eq (5). Then

$$\begin{array}{c} \log_{2}\left(m_{q}\left(X_{n}\right)\right)=-qHn+\log_{2}\left(m_{q}\left(X_{0}\right)\right) \end{array}$$ (6)

Also, from Eq (4), it holds that

$$\begin{array}{c} H=\frac{1}{q}\cdot \log_{2}\frac{m_{q}\left(X_{n}\right)}{m_{q}\left(X_{n+1}\right)}. \end{array}$$ (7)

The approach in Eq (6) for self-similarity index calculation purposes is named as FD algorithm. However, to properly apply the FD algorithm, it becomes necessary to guarantee the existence of *m*_*q*_(*X*). With this aim, FD4 algorithm stands by setting *q* = 0.01. In other words, FD4 approach is FD algorithm for *q* = 0.01. The reader may find a little bit artificial the selection of such a value for *q*. To justify that, we would like to point out that any *q* ≠ 0 can be chosen (at a first glance) to calculate the self-similarity index of unifractal processes provided that the corresponding sample moment exists. For Lévy stable motions, the sample moments *m*_*q*_(*X*_*n*_) may not exist for *q* > *q*_0_, unlike it happens for (fractional) Brownian motions. This is the reason why we take *q* = 0.01 for FD4 application purposes.

To calculate the self-similarity index via FD4, we can proceed by one of the two following ways:

- From Eq (6), we can determine *H* by the slope of a regression line comparing *n* vs. log_2_ *m*_*q*_(*X*_*n*_). Indeed, a linear regression coefficient close to 1 would yield that Eq (5) stands for such a random function.
- Let us consider Eq (7) for *q* = 0.01. Hence,
  $$\begin{array}{c} H=100\cdot \log_{2}\frac{m_{q}\left(X_{n}\right)}{m_{q}\left(X_{n+1}\right)}. \end{array}$$ (8)

This allows calculating *H* through the ratio between the moments of cumulative ranges of consecutive periods. It is worth mentioning that the calculation of *m*_*q*_(*X*_*n*_) lies on a sample of *X*_*n*_. Since the length of each sample of *X*_*n*_ is equal to 2^n^, the accuracy in the calculation of *m*_*q*_(*X*_*n*_) will improve as *n* increases. Therefore, the calculation of *m*_*q*_(*X*_*n*_) may be carried out via any of the approaches described below:

(i) Apply Eq (8) for the two greatest values of *n* available.
(ii) Consider all the values of *n* and calculate the ratios for all the pairs of consecutive moments.
(iii) For a time series (of log prices), let us divide it into 2^n^ non-overlapping blocks with lengths equal to $k=\frac{\text{length(series)}}{2^{n}}$. Then
  1. Calculate the range, *R*_*i*_, of each block ${ℬ}_{i}=\{B_{1},\ldots ,B_{k}\}$, i.e., let *R*_*i*_ = max{*B*_*j*_ : 1 ≤ *j* ≤ *k*} − min{*B*_*j*_ : 1 ≤ *j* ≤ *k*} for *i* = 1, …, 2^*n*^.
  2. Calculate $m_{q}(X_{n})=\frac{1}{2^{n}}\sum_{i=1}^{2^{n}}R_{i}^{q}$.
(iv) An alternative approach to (iii) can be developed in terms of overlapping blocks. To deal with, let *k* = length(series)/2^*n*^, i.e., the number of elements in each block. As such, given *n*, let us start each block at any index 0, 1, …, *k* − 1. This way, *k* distinct estimations for *m*_*q*_(*X*_*n*_) hold. Then calculate their mean.

Observe that to determine the range of each block ${ℬ}_{i}$ in expression (iii) (1), both the maximum and the minimum values of each period are considered. In financial series, such values are usually known for each trading period. Hence, the greatest value of *n* equals length(series), so each block would contain only one element. However, the range of that element (the maximum minus the minimum) can be still calculated.

It is worth pointing out that the FD approach described in this section becomes a generalization of the so-called Fractal Dimension algorithms (c.f. [49]) and GM2 approach (c.f. [27, 48]) to calculate the paramater of processes with stationary and self-affine increments (c.f. [47, Theorem 3.1]). The accuracy of FD approach for self-similarity exponent calculation purposes was analysed for Lévy stable motions and (fractional) Brownian motions with series lengths ranging from 2^5^ to 2^10^ data (c.f. [47, Section 5]).

## 3 Exploring the self-similarity exponent in BTC-USD series

In this section, we shall perform a dynamic evolution of the self-similarity index of BTC-USD daily returns through time by FD4 approach.

The empirical data used in this article can be described in the following terms. Let *X*(*t*) be the log of the daily prices of BTC-USD series in the period ranging from mid 2010 to early 2019. Thus, the length of such a time series equals the number of trading days in that period since each day is a trading day in the case of Bitcoin price series.

Next, we verify the two hypotheses required to the increments of BTC-USD series to properly apply FD4 algorithm for self-similarity exponent calculation purposes, i.e., that such increments are stationary and self-affine. Regarding the stationarity of the increments of our Bitcoin (log) price series, we have applied augmented Dickey-Fuller unit root test for a null hypothesis consisting of the increments of that series not being stationary. A p-value equal to 0.0 was obtained, so at a significance level of 0.05, it holds that those increments are stationary. On the other hand, we have empirically verified the linear relationship appeared in Eq (6), which describes the running of FD4 approach. In this way, we have dynamically calculated the correlation coefficent for daily returns of Bitcoin (log) price series in the period from 2012 to early 2019 by a sliding window with a length equal to 512 days and overlapping data. S1 Fig displays a dynamic evolution of the correlation coefficient of log_2_(*m*_*q*_(*X*_*n*_)) vs. *n*. S2 Fig shows that comparison for the last block of 512 data, which mainly describes the overall behavior of the series (in that case, the value of the correlation coefficient is equal to 0.99998). Finally, S3 Fig illustrates such a comparison for a block of 512 data ending in mid June 2012. In that occasion, the correlation coefficient was found to be equal to 0.988, which is among the lowest correlation coefficients for all the period analysed. Thus, the self-affine pattern of Bitcoin daily return series becomes quite strong and only that self-affine pattern would have been perturbed slightly at the beginning of the series.

The evolution of BTC-USD daily (log) prices is depicted in S4(a) Fig in the period ranging from 2012 to early 2019. In addition, the blue continous line in S4(b) Fig depicts a dynamic self-similarity exponent BTC-USD series by FD4 approach for a 512 day sliding window.

It is worth mentioning that overlapping data were considered for a more stably approach to the actual self-similarity exponent of BTC-USD series despite similar results were obtained by means of non-overlapping data.

Notice that S4(c) Fig highlights that all the self-similarity exponents of BTC-USD series stand above 0.5 in the whole period analysed (the red straight line marks the threshold of randomness), suggesting a persistent behavior of Bitcoin market in time.

However, does BTC-USD series behave inefficiently along from 2012 to early 2019? We should highlight here that a self-similarity exponent distinct from 0.5 is not necessarily due to a long range dependence in the series but also to the distribution of its increments (c.f., e.g., [27] for additional details). As such, to explore possible reasons why the self-similarity exponent of BTC-USD series stands above 0.5 in the whole period analysed, we shall proceed as follows.

Let *H*_*s*_ denote the self-similarity exponent of the shuffled BTC-USD series, i.e., the series generated from the BTC-USD one by randomly arranging its returns. This way, the shuffled series lacks the potential memory in the original BTC-USD series. Next, we define the *memory* indicator as the difference between the self-similarity exponent of the actual BTC-USD series and the self-similarity index of its shuffled series, i.e., let memory be equal to *H* − *H*_*s*_. In other words, if both *H* and *H*_*s*_ stand close, then we could infer that there is no memory in the series. Thus, if the self-similarity exponent of the BTC-USD series stands greater than 0.5, then it could be an effect of the distribution of its increments and not to the presence of memory. In this paper, we shall understand that market behaves efficiently provided that statistical arbitrage cannot be performed with the aim to gain market advantage based on information provided by indicators as the memory one analysed in this work.

S4(b) Fig depicts a dynamic evolution of both the self-similarity index of BTC-USD series (blue continuous line) and its shuffled series (black dotted line). The self-similarity exponents have been calculated via FD4 approach (*q* = 0.01) by a sliding window of size equal to 512 days with overlapping data.

Moreover, S4(c) Fig displays a dynamic evolution of the memory indicator (depicted by a blue continuous line) in time. Notice that the red straight line at height 0 means the absence of memory in the series. We used Montecarlo simulation, randomly shuffling the returns of the series of the 512 previous days for each week to obtain the distribution of *H*_*s*_. As such, we obtain the confidence intervals (at a confidence level of 90%) for each week, which are depicted in S4(c) Fig (black continuous lines). Thus, when *H* is out of the confidence intervals, we could conclude that there is either persistence (if *H* stands above the intervals) or anti-persistence (if *H* appears below the intervals) at such a confidence level.

Similar analyses have been carried out by calculating the self-similarity exponents of the series via FD4 approach for distinct sizes of sliding windows. First of all, it is worth pointing out that lower sizes for sliding windows lead to less accurate self-similarity exponents by FD4. This is the reason why we initially selected a 512 day sliding window to accurately calculate self-similarity exponents in BTC-USD series via FD4 algorithm. In addition, greater sizes of sliding windows allow a more stable evolution of that quantity through time. Following the above sliding windows with sizes equal to 64, 128, 256, and 1024 days were considered. The obtained results were found to be similar to those obtained for the case of a 512 day sliding window (c.f. S5, S6, S7 and S8 Figs).

Another option consists of calculating the self-similarity exponents of BTC-USD series by applying FD algorithm for other values of *q* distinct from 0.01. In this way, observe that the greater the value of *q*, the lower its corresponding self-similarity exponent, *H*(*q*). For illustration purposes, both S9 and S10 Figs depict the results obtained for *q* = 1 and *q* = 2 via FD algorithm by sliding windows with sizes equal to 512 days. The results were found to be similar to those provided by FD4 approach (*q* = 0.01). However, the self-similarity exponents do not always stand above 0.5 in the case of *q* = 2 (c.f. S10 Fig).

As a result, we obtained that the self-similarity exponent of the BTC-USD (log) price series stands above 0.5. However, this is not due to the presence of significant memory in the series but to its underlying distribution. In fact, it holds that the self-similarity exponent of BTC-USD price series is similar or lower than the self-similarity index of a random series with the same distribution, with several periods with significant antipersistent memory in BTC-USD price series.

Finally, we have also explored the dynamic evolution of the self-similarity exponent of actual S&P500 series in the same time period. To deal with, FD4 approach for *q* = 0.01 has been applied by sliding windows with sizes equal to 256 and 512 days, respectively. The obtained results (c.f. both S11 and S12 Figs) are quite similar to the results obtained by analysing the evolution of the memory indicator in the BTC-USD series (by using different sizes of the sliding windows). More specifically, we found that the self-similarity exponent of the S&P500 series (in log format) stands greater than 0.5 in the whole period analysed. But this is not due to the presence of significant memory in the series but to its underlying distribution. As such, we conclude that there are several periods with significant antipersistent memory in S&P500 price series.

## 4 Conclusions

In this paper, we have explored the (in)efficiency of the continuum Bitcoin (BTC-USD) market from mid 2010 to early 2019. With this aim, we have dynamically analysed the evolution of the self-similarity exponent of BTC-USD daily (log) prices via FD4 approach by a 512 day sliding window with overlapping data. We would like to highlight that we are not assuming any specific model in regard to the underlying distribution of BTC-USD (resp., S&P500) series beyond the two hypotheses that have been verified to properly apply FD algorithm. It was found that the self-similarity exponent of BTC-USD market stands above 0.5 in the whole period analysed. This throws some empirical evidence concerning a persistent behavior of that series.

Some reasons for such a persistency have been explored. To deal with, the *memory* indicator has been defined as the difference between the self-similarity exponent of BTC-USD series and the self-similarity index of its shuffled series, i.e., the series obtained by randomly arranging the returns of the original BTC-USD series.

Similar analyses to explore the dynamic evolution of the self-similarity exponents of BTC-USD series have been carried out via FD4 approach by sliding windows with sizes equal to 64, 128, 256, and 1024 days. The obtained results were found to be quite similar to the case of a 512 day sliding window. Moreover, all the calculations have been also developed by FD algorithm for values of *q* equal to 1 and 2 and 512 day sliding windows, as well. We observe that the greater the value of *q*, the lower its corresponding self-similarity exponent, *H*(*q*), and hence, the self-similarity exponents of both BTC-USD series and its shuffled series do not always stand above 0.5.

We conclude that the self-similarity exponent of the BTC-USD (log) price series stands distinct from 0.5. However, this is not due to the presence of significant memory in the series but to its underlying distribution. In fact, it holds that the self-similarity exponent of BTC-USD series is similar or lower than the self-similarity index of a random series with the same distribution, and there are several periods with significant antipersistent memory in BTC-USD series.

Finally, we have compared the results obtained for BTC-USD series with respect to the ones from actual S&P500 index in the same period, from mid 2010 to early 2019. With this aim, FD4 approach for *q* = 0.01 has been applied by sliding windows with sizes equal to 256 and 512 days, respectively. The obtained results were found to be quite similar to the results obtained by analysing the evolution of the memory indicator in the BTC-USD series (by different sizes of the sliding windows). More specifically, we found that the self-similarity exponent of the S&P500 series (in log format) stands greater than 0.5 in the whole period analysed. But this is not due to the presence of significant memory in the series but to its underlying distribution. As such, we conclude that there are several periods with significant antipersistent memory in S&P500 price series.

## Supporting information

S1 File BTC-USD daily price series from mid 2010 to early 2019. (CSV)

S2 File S&P500 daily price series from mid 2010 to early 2019. (CSV)

S3 File Source code of the FD4 approach as written by MA Sánchez. (RTF)

S1 Fig Dynamic evolution of the correlation coefficient of log_2_(*m*_*q*_(*X*_*n*_)) vs. *n*. (TIFF)

S2 Fig The overall behavior of the series is characterized by a strong self-affinity pattern. (TIFF)

S3 Fig The beginning of the series slightly disturbs the self-affine pattern of the Bitcoin daily return series. (TIFF)

S4 Fig (a) BTC-USD daily (log) prices in the period ranging from 2012 to early 2019. (b) They have been depicted a dynamic self-similarity exponent of BTC-USD series (blue continuous line) together with its shuffled series (black dotted line). (c) Dynamic evolution of the memory indicator (depicted by a blue continuous line) in time. The corresponding confidence intervals (at a confidence level of 90%) have been plotted by black continuous lines. The self-similarity exponents have been calculated via FD4 approach (*q* = 0.01) by a sliding window of size equal to 512 days with overlapping data.
(TIFF)

S5 Fig Similar analysis to the one provided in S4 Fig via FD4 approach (*q* = 0.01) by a sliding window of size equal to 64 days. (TIFF)

S6 Fig Similar analysis to the one provided in S4 Fig via FD4 approach (*q* = 0.01) by a sliding window of size equal to 128 days. (TIFF)

S7 Fig Similar analysis to the one provided in S4 Fig via FD4 approach (*q* = 0.01) by a sliding window of size equal to 256 days. (TIFF)

S8 Fig Similar analysis to the one provided in S4 Fig via FD4 approach (*q* = 0.01) by a sliding window of size equal to 1024 days. (TIFF)

S9 Fig Similar analysis to the one provided in S4 Fig via FD approach (with *q* = 1) by a sliding window of size equal to 512 days. (TIFF)

S10 Fig Similar analysis to the one provided in S4 Fig via FD approach (with *q* = 2) by a sliding window of size equal to 512 days. (TIFF)

S11 Fig Analysis of significant memory (at a confidence level of 90%) in actual S&P500 series for the period ranging from 2012 to early 2019 via FD4 algorithm (*q* = 0.01) by a sliding window of size equal to 256 days. (TIFF)

S12 Fig Similar analysis to the one provided in S11 Fig via FD4 approach (*q* = 0.01) by a sliding window of size equal to 512 days. (TIFF)
