# Supplementary material for: Some comments on Bitcoin market (in)efficiency
Source: PLoS One. 2019 Jul 8;14(7):e0219243. doi: 10.1371/journal.pone.0219243 (PMC6613746; doi:10.1371/journal.pone.0219243)
Supplement: S3 File — (RTF) [file pone.0219243.s003.rtf]

#Python codefrom bumpy import log, log2, sumfrom spicy.stats import linregressdef fd4(high,low,q=0.01,nmin=0,nmax=-2):    """Calculate the Hurst Exponent H using FD algorithm with overlapping windows	params:		high: maximum of the series each period. For example, the logarithm of the high of a stock each day		low: minimum of the series each period. For example, the logarithm of the low of a stock each day		q: exponent of multifractal analysis. For FD4 use q=0.01		nmin: the length of the minimum window used will be 2**nmin		nmax: the length of the maximum window used will be 2**nmax. nmax==-2 means that the maximum window used will be a quarter of the length of the series, nmax=-1 means that the maximum window used will be half of the length of the series.	remarks:		You will usually use the default values for nmin and nmax and often for q, too.		high and low are series of the same length	reference:		M. Fernandez-Martinez, M.A. Sanchez-Granero, J.E. Trinidad Segovia, I.M. Roman Sanchez. An accurate algorithm to calculate the Hurst exponent of self-similar processes, Physics Letters A, 378 (2014), 2355-2362.”””    def k(high,low,q,tau):        aux=[]        for i in range(len(serie)-tau+1):            maximo=log(max(serie.high[i:i+tau]))            minimo=log(min(serie.low[i:i+tau]))            aux.append((maximo-minimo)**q)        return sum(aux)/(len(serie)-tau+1.0)    nmax=int(log2(len(high)))+nmax    rango=[2**n for n in range(nmin,nmax)]    kq=[k(high,low,q,tau) for tau in rango]    b1=log(rango)    b2=log(kq)    lr=linregress(b1,b2)    return lr[0]/q
