# Supplementary figures and images for: Some comments on Bitcoin market (in)efficiency
Source: PLoS One. 2019 Jul 8;14(7):e0219243. doi: 10.1371/journal.pone.0219243 (PMC6613746; doi:10.1371/journal.pone.0219243)

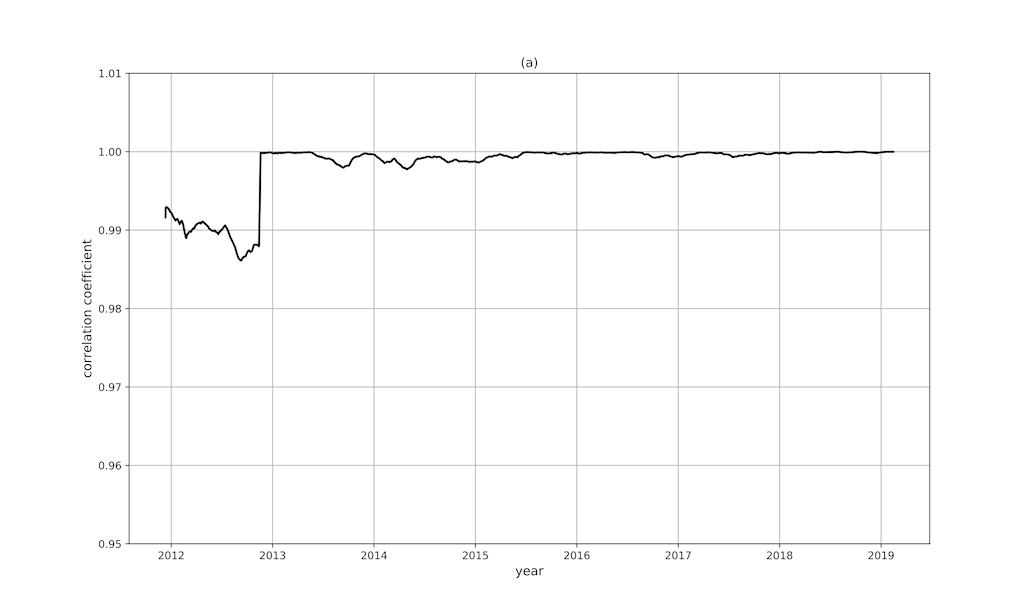

Supplement: S1 Fig — (TIFF) [file pone.0219243.s004.tiff]

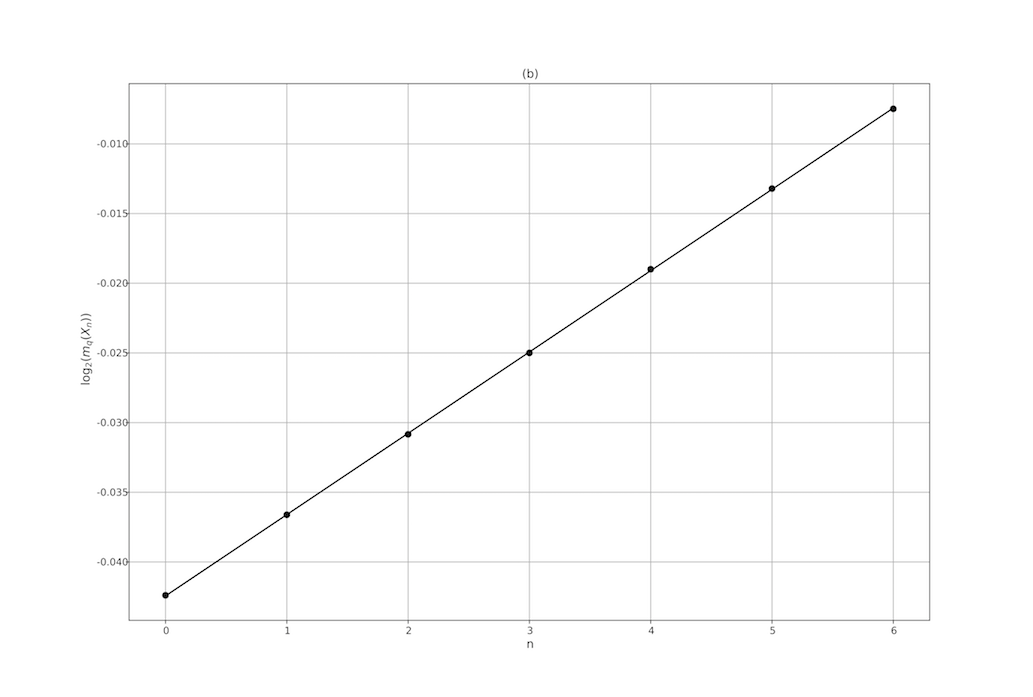

Supplement: S2 Fig — (TIFF) [file pone.0219243.s005.tiff]

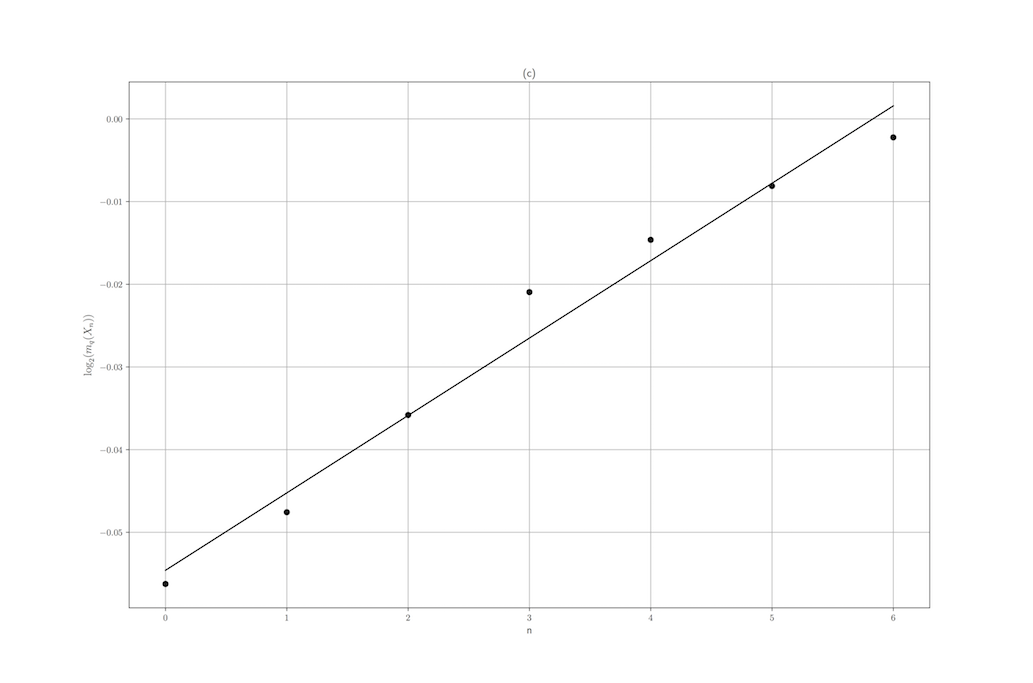

Supplement: S3 Fig — (TIFF) [file pone.0219243.s006.tiff]

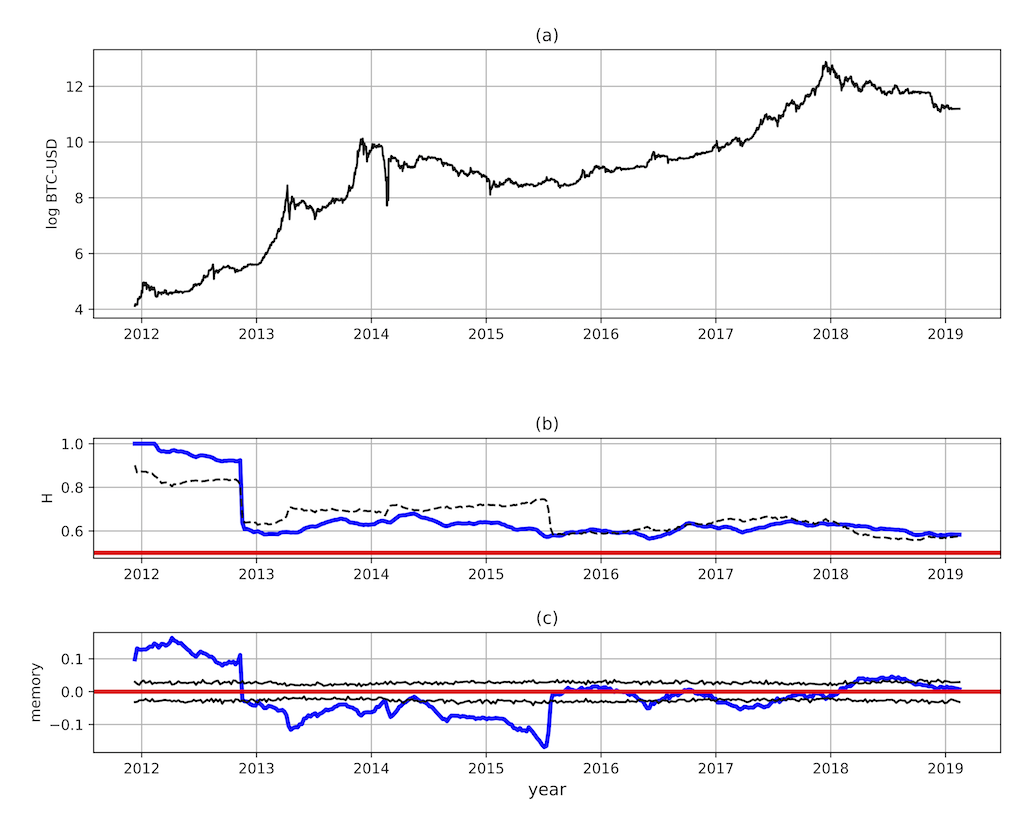

Supplement: S4 Fig — (a) BTC-USD daily (log) prices in the period ranging from 2012 to early 2019. (b) They have been depicted a dynamic self-similarity exponent of BTC-USD series (blue continuous line) together with its shuffled series (black dotted line). (c) Dynamic evolution of the memory indicator (depicted by a blue continuous line) in time. The corresponding confidence intervals (at a confidence level of 90%) have been plotted by black continuous lines. The self-similarity exponents have been calculated via FD4 approach (q = 0.01) by a sliding window of size equal to 512 days with overlapping data. (TIFF) [file pone.0219243.s007.tiff]

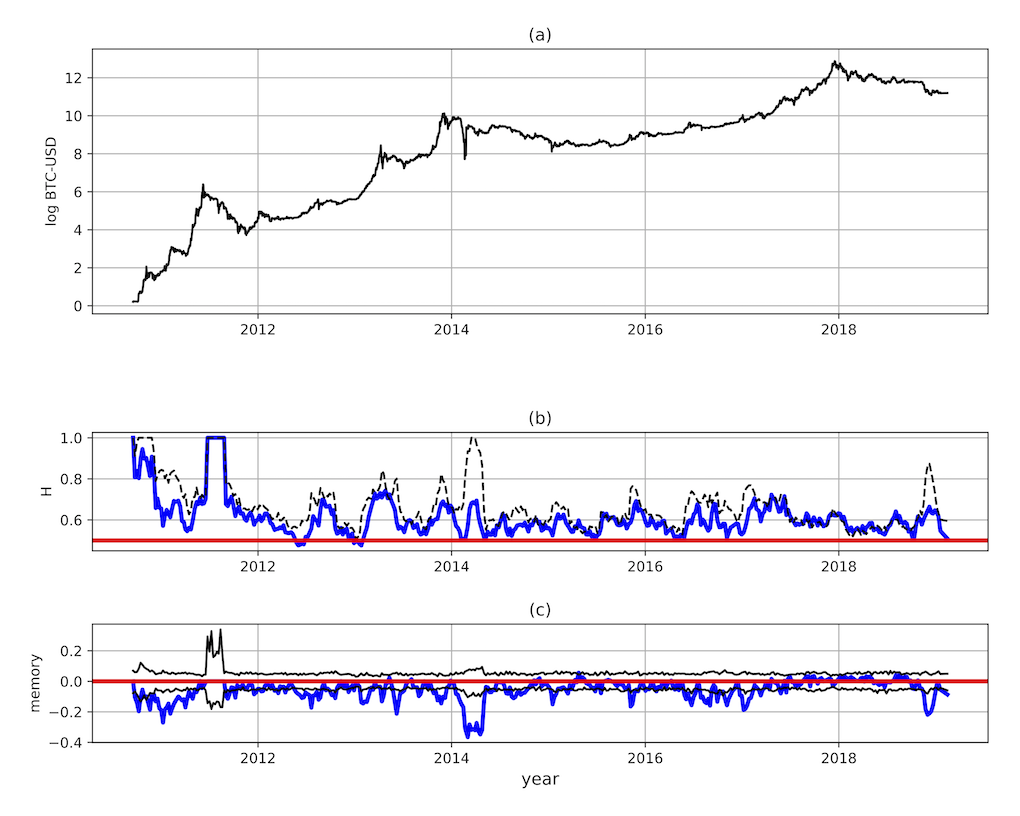

Supplement: S5 Fig — (TIFF) [file pone.0219243.s008.tiff]

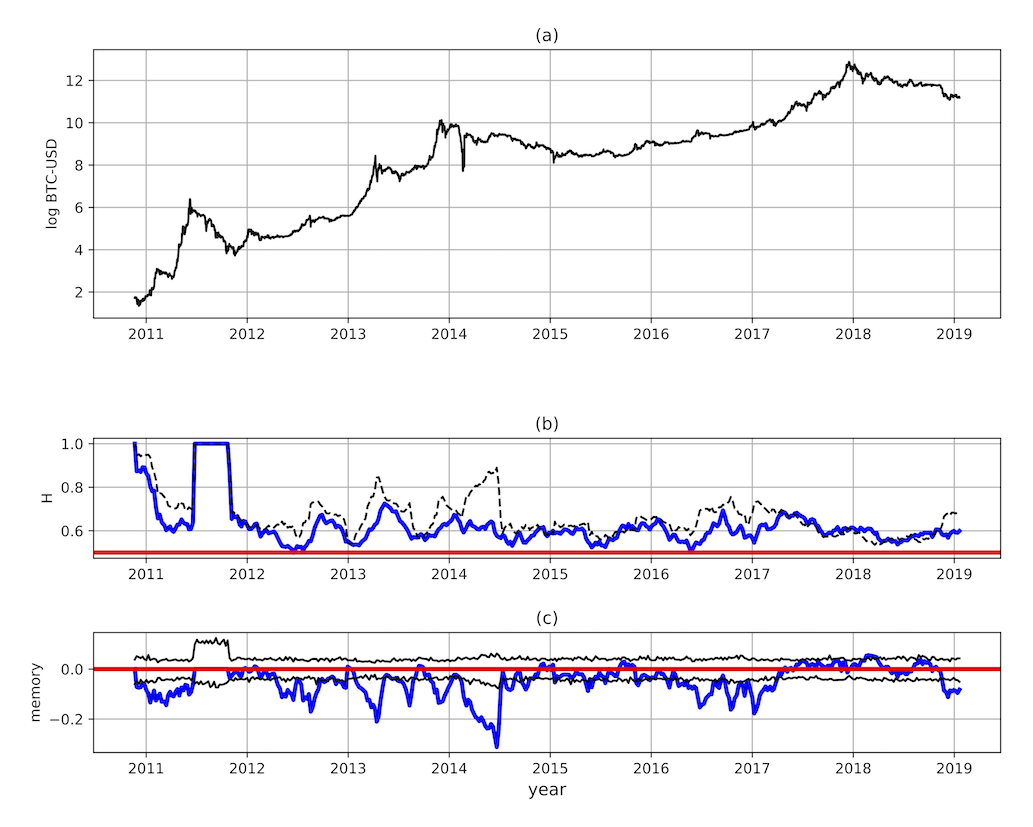

Supplement: S6 Fig — (TIFF) [file pone.0219243.s009.tiff]

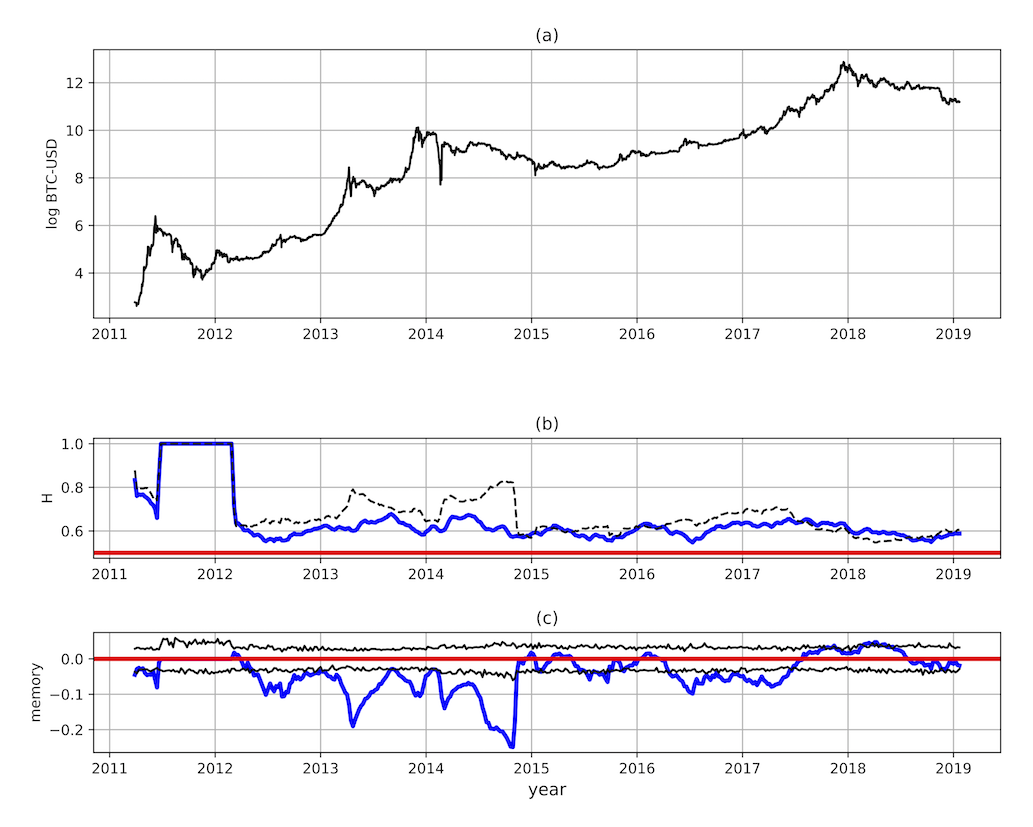

Supplement: S7 Fig — (TIFF) [file pone.0219243.s010.tiff]

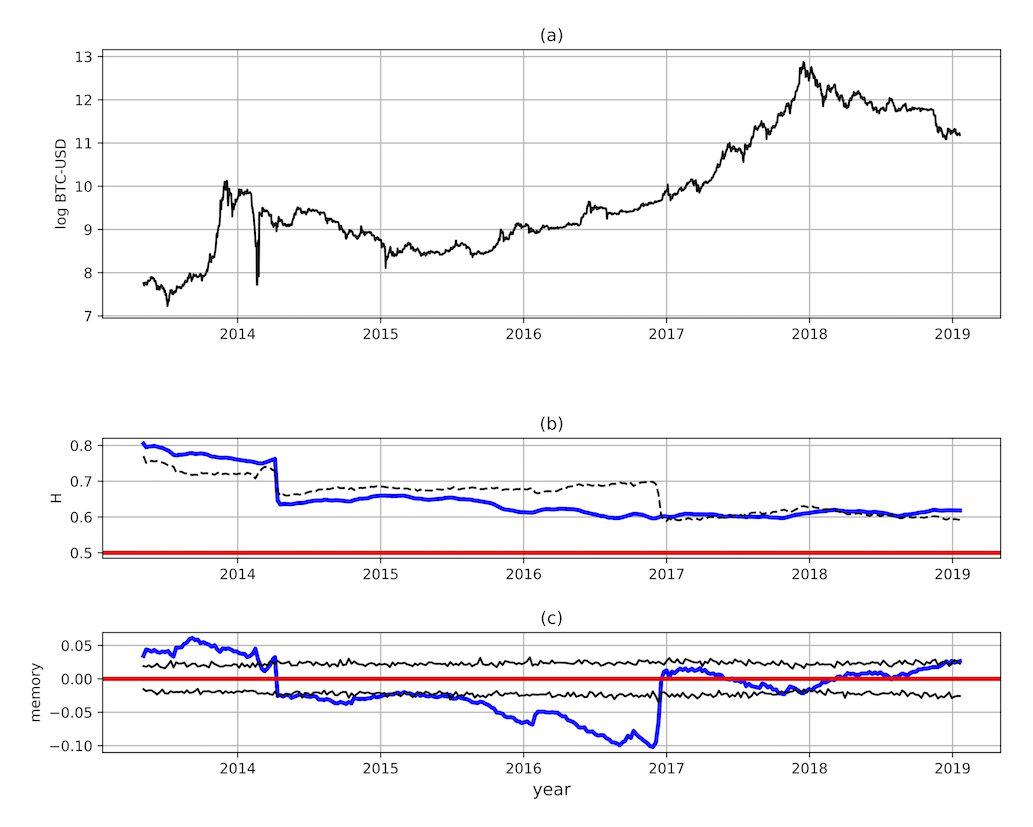

Supplement: S8 Fig — (TIFF) [file pone.0219243.s011.tiff]

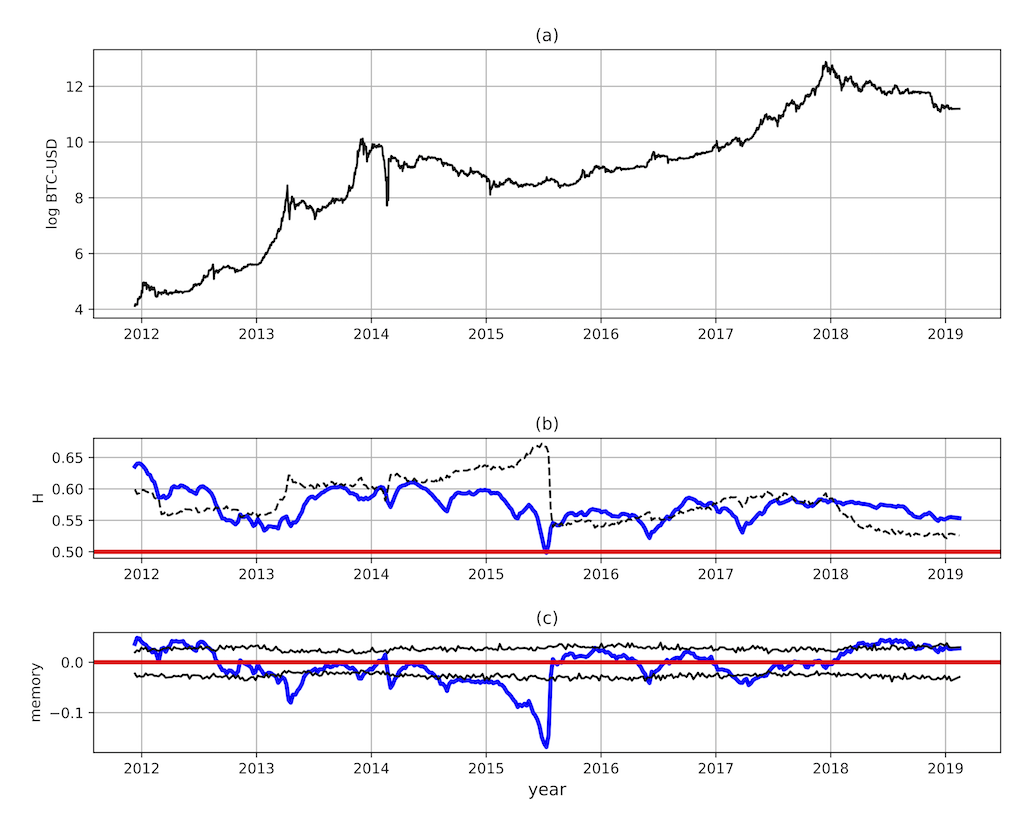

Supplement: S9 Fig — (TIFF) [file pone.0219243.s012.tiff]

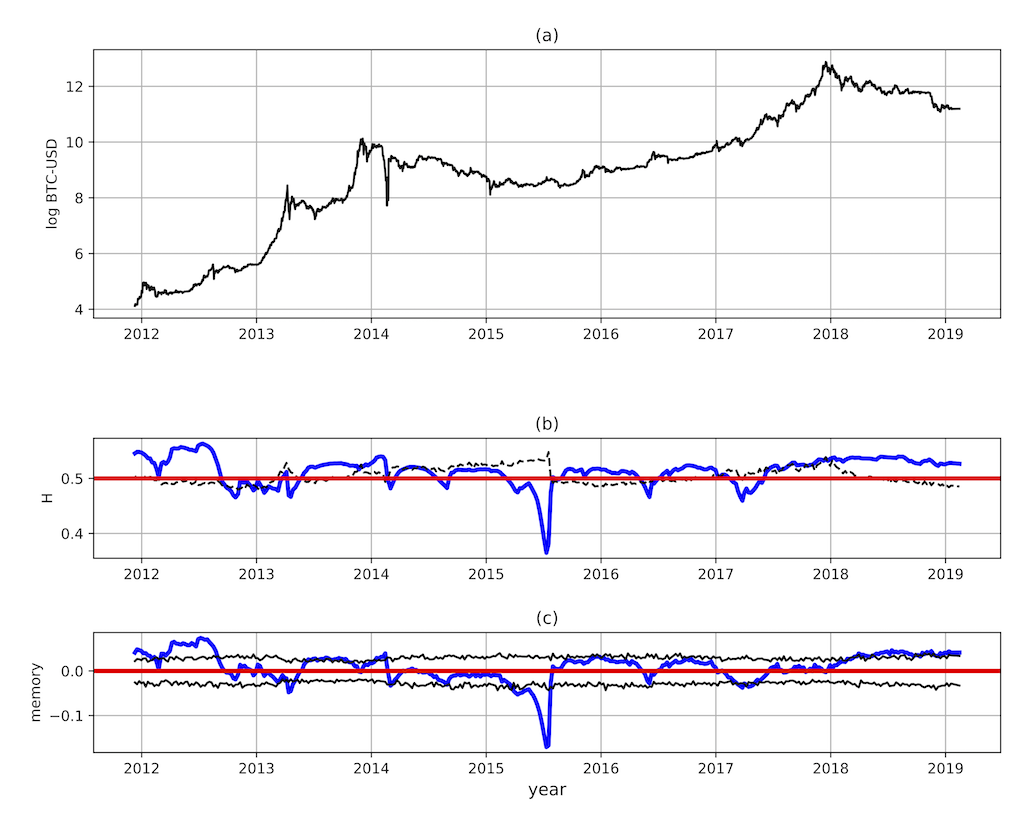

Supplement: S10 Fig — (TIFF) [file pone.0219243.s013.tiff]

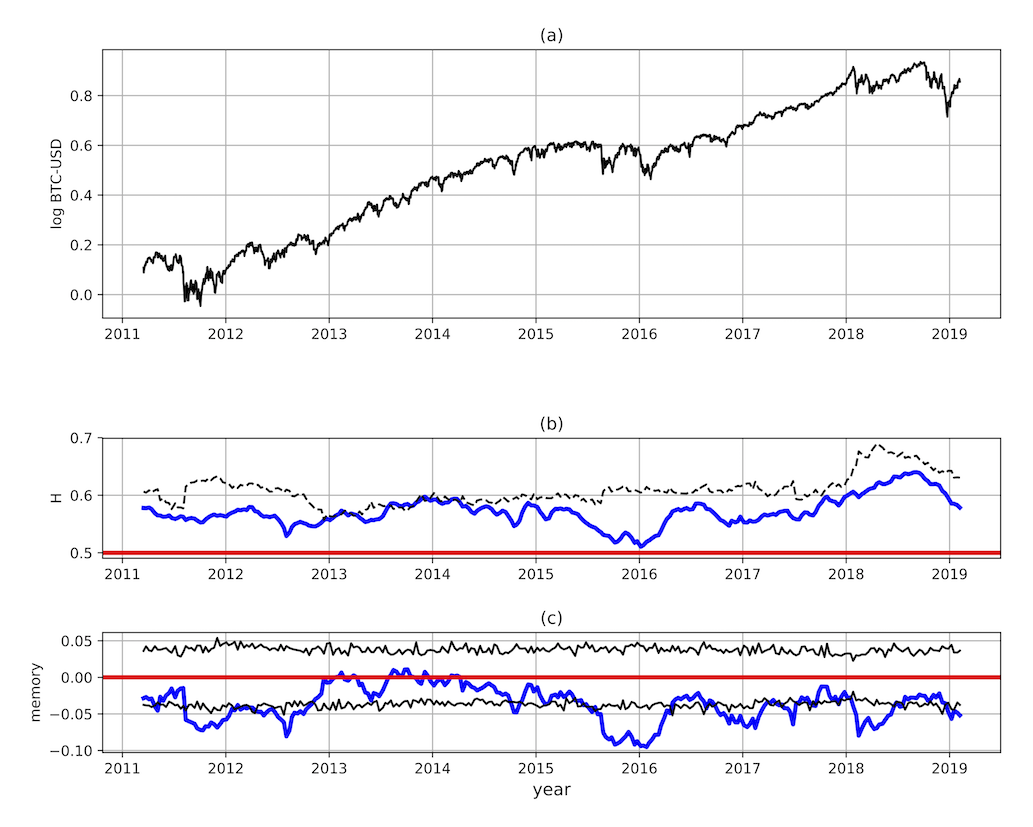

Supplement: S11 Fig — (TIFF) [file pone.0219243.s014.tiff]

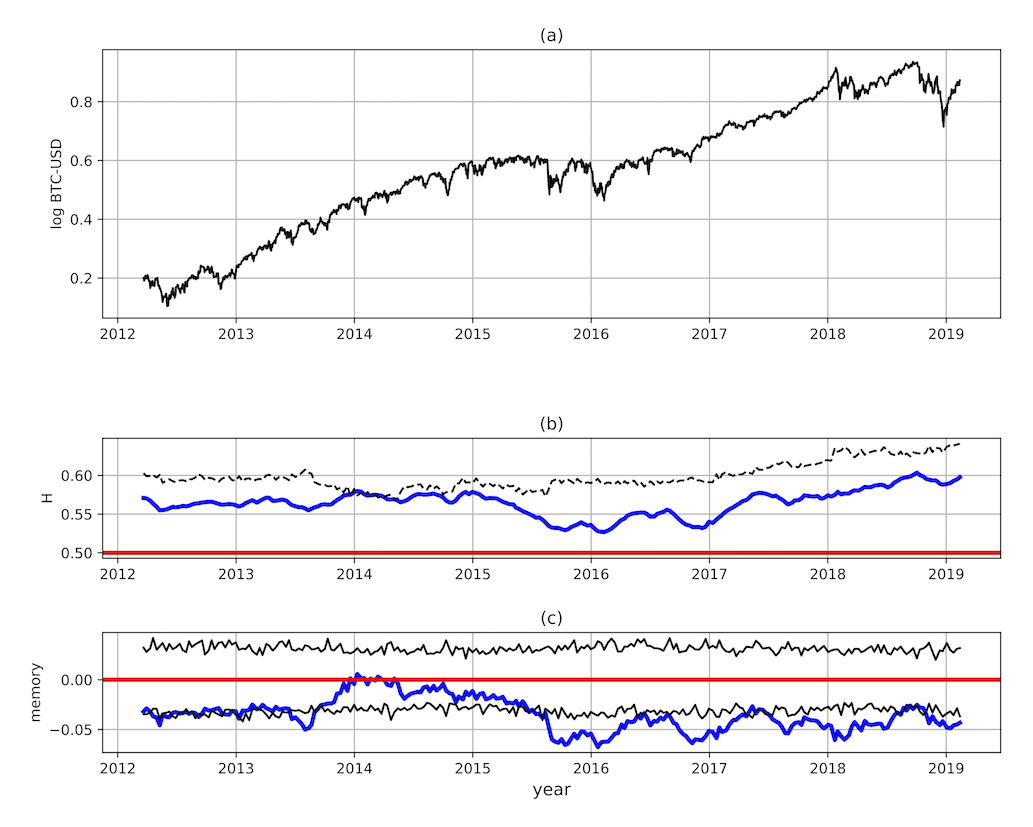

Supplement: S12 Fig — (TIFF) [file pone.0219243.s015.tiff]
